# Treatment Mechanism of *Gardeniae* Fructus and Its Carbonized Product Against Ethanol-Induced Gastric Lesions in Rats

**DOI:** 10.3389/fphar.2019.00750

**Published:** 2019-07-03

**Authors:** Xue Zhang, Yun Wang, Xiaoqing Li, Yejia Dai, Qinghao Wang, Guoyou Wang, Depeng Liu, Xuezhu Gu, Dingrong Yu, Yinlian Ma, Cun Zhang

**Affiliations:** ^1^Institute of Chinese Materia Medica, China Academy of Chinese Medical Sciences, Beijing, China; ^2^College of Pharmacy, Henan University of Chinese Medicine, Zhengzhou, China; ^3^College of Pharmacy, Anhui University of Chinese Medicine, Hefei, China

**Keywords:** *Gardeniae* Fructus, processed, carbonized *Gardeniae* Fructus, ethanol-induced gastric lesion, metabolomics, gastroprotective

## Abstract

*Gardeniae* Fructus (GF) and carbonized GF (GFC) have been shown to exert a gastrointestinal protective effect and are frequently used in clinical practice for the treatment of hemorrhage and brown stool. In this study, we employed a combination of pharmacological methods and metabolomics in a rat model of ethanol-induced acute stomach ulcer to investigate the gastroprotective effect of GF and GFC water extracts and the potential mechanism involved in this process. The levels of nitric oxide (NO) and interleukin 6 (IL-6) in the plasma of rats were determined. The results showed that both GF and GFC reduced the ethanol-induced gastric lesions and expression of NO and IL-6 in these rats. Of note, 16 and 11 feature metabolites were filtered and identified in the GF and GFC groups, respectively. Both GF and GFC act by restoring the biosynthesis of valine, leucine, and isoleucine, and the metabolism of glycerophospholipids. Moreover, histological evaluation revealed that heat processing of GF to create GFC enhanced the gastric mucosa protective effect. Furthermore, heat processing converted the main pathway from alanine, aspartate, and glutamate metabolism, associated with GF, to histidine metabolism, associated with GFC. GF and GFC ameliorated gastric mucosa lesions in rats *via* reductions in NO production and inflammatory cytokine secretion, and the induction of prostaglandin E2.

## Introduction

Hematemesis and melena or hematochezia are signs and symptoms of acute upper gastrointestinal bleeding (AUGIB). Despite a deceasing trend in the overall incidence of AUGIB observed over the previous 30 years (Targownik and Nabalamba, 2006; Theocharis et al., 2008; Rotondano, 2014), it remains one of the most common gastrointestinal emergencies worldwide. A multicentre survey involving 6,750 patients investigated AUGIB in the United Kingdom. The results showed that the presence of peptic ulcers played an important role in the induction of hemorrhage, accounting for 36% of cases (Hearnshaw et al., 2011). Pharmacotherapy is currently the main approach for the treatment of these low-risk patients and the use of potent acid-suppressing proton-pump inhibitors has been recommended (Gralnek et al., 2008). Recently, natural products with antioxidant and gastroprotective properties have attracted the attention of complementary doctors and been used in the treatment of peptic ulcers which may lead to non-variceal upper gastrointestinal hemorrhage (Repetto and Llesuy, 2002; Awaad et al., 2013). However, such products have been in traditional Chinese medicine (TCM) for thousands of years in the treatment of similar syndromes to AUGIB. One of the most common reasons for the development of AUGIB is the presence of a peptic ulcer. A nationwide population-based study, involving 1 million individuals, found that 15.5% patients diagnosed with a peptic ulcer were treated through TCM (Huang et al., 2015). *Gardeniae* Fructus (GF) and its heat-processed products are commonly used in treatment of hematemesis and hematochezia.

A search in the Encyclopedia of Chinese Medicine—containing 1,156 ancient Chinese medical books—revealed that GF and carbonized GF (GFC) were frequently used in the treatment of hemorrhage and brown stool. Processing of natural products is a feature of TCM to fulfill different requirements of therapy (Zhao et al., 2010). One of the aims of using processed GF is to reduce side effects to patients with stomach weakness. For example, the first use of the ‘Huanglian Jiedu’ decoction to drain fire and vomiting blood in the form of GF was recorded in ‘Zhou Hou Fang’. Moreover, a pediatric disease book titled ‘You You Ji Cheng’ described the treatment of hematemesis and post-defecation bleeding in children using with GFC in the ‘Huanglian Jiedu’ decoction. Nowadays, chemical research has been conducted to study the active substances in GF. Researchers found that the chemical composition of GF includes iridoid glycosides (Zhang et al., 2009a; Wang et al., 2015), phenylpropanoids (Zuo et al., 2015), crocins (Zhang et al., 2009b), glycoprotein (Lu et al., 2018), and polysaccharides, etc. Among those, iridoids and crocins are the two main constituents of GF, the levels of which markedly decreased after heat processing (Hong, 2007; Jian et al., 2015; Ling et al., 2017). Meanwhile, after heat processing, the tannin content of GFC increased (Lan et al., 2014; Ling et al., 2017). Pharmacodynamic experiments showed that the water extract of GFC may reduce the secretion of gastric acid (Zhang et al., 1994), promote pepsin activity (Zhang et al., 1994), and shorten the blood clotting time in mice (Ping et al., 2008; Lan et al., 2014). In addition, the ethanol extract of GF may reduce the risk of gastritis and gastric lesions, similarly to ursolic acid and genipin (Lee et al., 2009). Therefore, we hypothesized that the water extract of GF and GFC may ameliorate peptic ulcers for the treatment of hematemesis in the clinic.

To test this hypothesis, we combined pharmacological methods and metabolomics in a rat model of ethanol-induced acute stomach ulcers. A review of articles discussing the gastroprotective effects of natural products in animal models of ethanol-induced acute peptic ulcers (Matsumoto et al., 2004; Park et al., 2008; Al Batran et al., 2013; Saeed Al-Wajeeh et al., 2016) showed that ethanol induced gastric hemorrhagic erosions (Szabo et al., 1985). And papers showed some of constituents in herbs could significantly reduced the pro-inflammatory cytokine interleukin 6 (IL-6), Tumor necrosis factor alpha (TNF-α) and have the ability to attenuate inflammation by targeting different intracellular signaling pathways triggered by nuclear factor-kappa B (NF-κB) and MAPKs etc (Chen et al., 2018; Chen et al., 2019). Nowadays, metabolomics are widely used to discriminate the profiles of chemical constituents and reveal the mechanism of action involved in herb processing. For example, metabolomics studies using reverse-phase liquid chromatography quadrupole-time of flight (RPLC-Q-TOF)/Mass Spectrometry (MS) to investigate chemical markers after processing of Radix aconite (Li et al., 2013), and the potential treatment mechanism of GF against type 2 diabetes in rats *via* targeted metabolome profiling and fecal metabolomics (Wang et al., 2017; Zhou et al., 2018). In this study, we initially used a rat model of ethanol-induced acute stomach ulcer treated with GF and GFC. Subsequently, we selected the antioxidant/anti-inflammation index and histology of gastric lesions to evaluate the gastroprotective effect of GF and GFC. Based on the pharmacological effect, we analyzed the plasma of rats using hydrophilic interaction liquid chromatography (HILIC)/ultra high performance liquid chromatography (UHPLC)/MS and C18/UHPLC/MS to obtain their metabolomics profile. Finally, multivariate data and pathway analyses were used to investigate the treatment mechanism of GF and GFC.

## Materials and Methods

### Plant Specimens and Preparation of Extract

Samples of GF and GFC were came from same path of pieces, and processed in Baicaokangshen Pharmaceutical Co., Ltd. (Hebei, China). The GFC was processed as black outside and brown inside with strong fire according to the Standard for Processing of Chinese Herbal Medicine Slices in China. The GF and GFC were authenticated by Prof. Zhang Cun to be the dry fruit and fried charcoal product of *Gardenia jasminoides* Ellis. The specimens of GF and GFC were deposited at the Herbarium of National Resource Centre for Chinese Materia Medica, China Academy of Chinese Medical Sciences, with the following reference numbers: CMMIYC-04639 and CMMIYC-04641. GF and GFC were extracted twice (20 min per extraction) using boiled water. The water extracts of GF and GFC were converted into freeze-dried powder after vacuum evaporation. The content of gardoside; genipin 1-gentiobioside, geniposide, p-coumaroylgenipin gentiobioside, crocin I, and crocin II of GF and GFC were determined by HPLC (Zhang et al., 2019). The results are shown in the **Supplementary Figure 1** and **Supplementary Table 1**.

### Animal Model

Male Sprague–Dawley (weight: 180–200 g) rats were purchased from Charles River (Beijing Vital River Laboratory Animal Technology Co., Ltd., Beijing, China). The animals were housed in an isolated room maintained at 24 ± 1°C and 55 ± 10% relative humidity with an equal 12-h light-dark cycle, under good laboratory practice conditions according to the Chinese Academy of Chinese Medical Sciences. The rats were randomly classified into four groups (six rats per group). Rats in the GF and GFC groups received oral gavage with freeze-dried power of GF and GFC with 0.5% CMC-Na at a dose of 4.5 g·kg^−1^ for 7 days (Bei-hua et al., 2013; Chen et al., 2014). On the 7^th^ day, absolute ethanol (5 ml·kg^−1^) was administered orally to the rats in the ulcer model (M), GF, and GFC groups. An equal volume of saline was administered to the normal control (NC) group. After 60 min, the rats of the GF and GFC groups were treated with water extract at a dose of 4.5 g·kg^−1^ (10 ml·kg^−1^) (Golbabapour et al., 2013). The rats of the NC and M groups were treated with an equal volume of saline. The animals were anaesthetized 60 min after treatment with 10% chloral hydrate (3.5 ml·kg^−1^) and their blood and stomachs were immediately collected.

### Gastroprotective Effects

#### Antioxidant Activity

The blood was immediately centrifuged at 3,500 rpm for 15 min at 4°C and the serum (supernatant) was collected according to the instructions of the manufacturer of nitric oxide (NO) and IL-6 (Nanjing Jiancheng Bioengineering Institute, Nanjing, China). The levels of NO and IL-6 in the serum were measured *via* the nitrate reductase and enzyme-linked immunosorbent assay method (Almasaudi et al., 2016; Liu et al., 2017).

#### Histology of Gastric Lesions

After sacrificing the animals, the stomachs were removed and washed with 0.9% saline solution. The areas of stomach lesions were measured using a ruler and the stomachs were transferred in buffered formalin solution for 48 h. Subsequently, the stomachs were removed from the formalin solution, washed with water, dehydrated with gradient ethanol, and embedded in paraffin blocks. Sections of 5 μm were stained with hematoxylin and eosin (H&E) and observed for pathological changes using ordinary light microscopy. The different degrees of damage to the gastric mucosa of rats were evaluated as follows: ‘+’ small mucosa segmental bleeding and normal muscularis mucosa; ‘++’ mucosa segmental hemorrhage with mild lesion of muscularis externa; ‘+++’ gastric mucosa with segmental hemorrhage, muscularis externa edema with infiltration of inflammatory cells.

### Metabonomic Study

#### Pre-treatment of Plasma

The blood was treated with heparin sodium and centrifuged at 3,500 rpm for 15 min to obtain the plasma for metabolomics analysis. Both HILIC and C18 were used to separate the plasma of rats in this study. For HILIC separation, 100 μl of plasma were treated with 300 μl acetonitrile and centrifuged at 14,000×g for 10 min to obtain the supernatant. For C18 separation, 100 μl of plasma were treated with 300 μl menthol and centrifuged at 14,000×g for 10 min. For the two separation modes, 100 μl of supernatant were analyzed.

#### UHPLC/MS Analysis

Chromatography was performed with an UltiMate™ 3000 Rapid Separation LC system (Thermo Scientific™, MA, USA). The flow rate was set at 300 µl·min^−1^ and the injection volume of the sample was 1 µl. The C18 separation was performed with a CSH C18 column (2.1 × 100 mm, 1.7 µm, Waters) operated at 45°C with a gradient elution program. The mobile phase consisted of acetonitrile/water (60/40, A) and isopropanol/acetonitrile (90/10, B) with 0.1% formic acid and 10 mmol·L^−1^ ammonium acetate. The gradient elution program proceeded as follows: 0–1 min, 20%B; 1–11 min, 20–100%B; 11–18 min, 100%B; and 18–19.5 min, 100–20%B. In HILIC separation, a Ethylene Bridged Hybrid (EBH) amide column (2.1 × 100 mm, 1.7 µm, Waters) and gradients of acetonitrile (mobile phase A) and water (mobile phase B) containing 0.1% formic acid and 10 mmol·L^−1^ ammonium acetate each were used to separate the plasma samples. The column was operated at 40°C. The gradient program was set as follows: 0–1 min, 5%B; 1–7 min, 5–50%B; and 7–12 min, 50%B.

The eluate from the LC system was introduced directly into the mass spectrometer (Q Exactive^™^, Thermo Scientific^™^, MA, USA) equipped with a HESI-II probe for detection, which was operated in positive mode. The positive HESI-II spray voltages were 3.7 kV, the heated capillary temperature was 320°C, the sheath gas pressure was 30 psi, the auxiliary gas setting was 10 psi, and the heated vaporizer temperature was 300°C. Data were collected in auto gain control under 1 × 10^6^ with a scan range of 150–1,500 mass-to-charge *m/z*. A maximum isolation time of 50 ms was used and the calibration was customized for the analysis of Q Exactive^™^ to maintain a mass tolerance of 5 ppm. Samples were analyzed in a single batch in random order with quality control samples (QCs). The LC-MS system was controlled using the Xcalibur 2.2 SP1.48 software (Thermo Scientific^™^, MA, USA), and data were collected and processed using the same software. The QCs involved a mixture of plasma samples from each sample, which were used to evaluate the stability of the LC/MS system. Feature ions with a coefficient of variation (CV) beyond 15% were removed.

#### Data Analysis

The raw data obtained from the LC/MS was processed using the Progenesis QI data analysis software (Newcastle, UK) for peak alignment, picking, and normalization to produce peak intensities for retention time (t_R_) and *m/z* data pairs. Multivariate data analysis was used to analyze the metabolomics data using the SIMCA-p 14.0 (Umeå, Sweden). Using Pareto scaling (par) to transform raw data to normally distributed prior to analysis. Subsequently, principal components analysis (PCA) was performed using the SIMCA-P software (version 14.0, Umetrics AB, Umeå, Sweden) to assess the main sources of variation and remove the sample beyond the confidence interval (95%). Subsequently, the feature ions were filtered through orthogonal partial least squares discriminant analysis (OPLS-DA). The ions with high reliability and magnitude were considered the putative metabolites in the S-plot based on the OPLS-DA models.

For the identification of potential biomarkers, molecular formulas were assessed by matching accurate *m/z* measurements to metabolites from the available online databases (http://www.hmdb.ca/ and http://www.lipidmaps.org/). Finally, we used the available online databases KEGG (http://www.genome.jp/kegg/) and MetaboAnalyst (https://www.metaboanalyst.ca/) for candidate compound pathway analysis.

#### Other Statistical Analyses

All data are presented as mean ± standard deviation. Weighing frequency cased, and the rank-sum test was used to compare the histopathological changes between the M and groups treated with drug. The statistical significance (*P* < 0.05 or *P* < 0.01) of differences between mean values was tested using the Student’s *t*-test.

## Results

### Evaluation of Gastroprotective Effects

#### Histological Evaluation of Gastric Lesions

**Figure 1:**
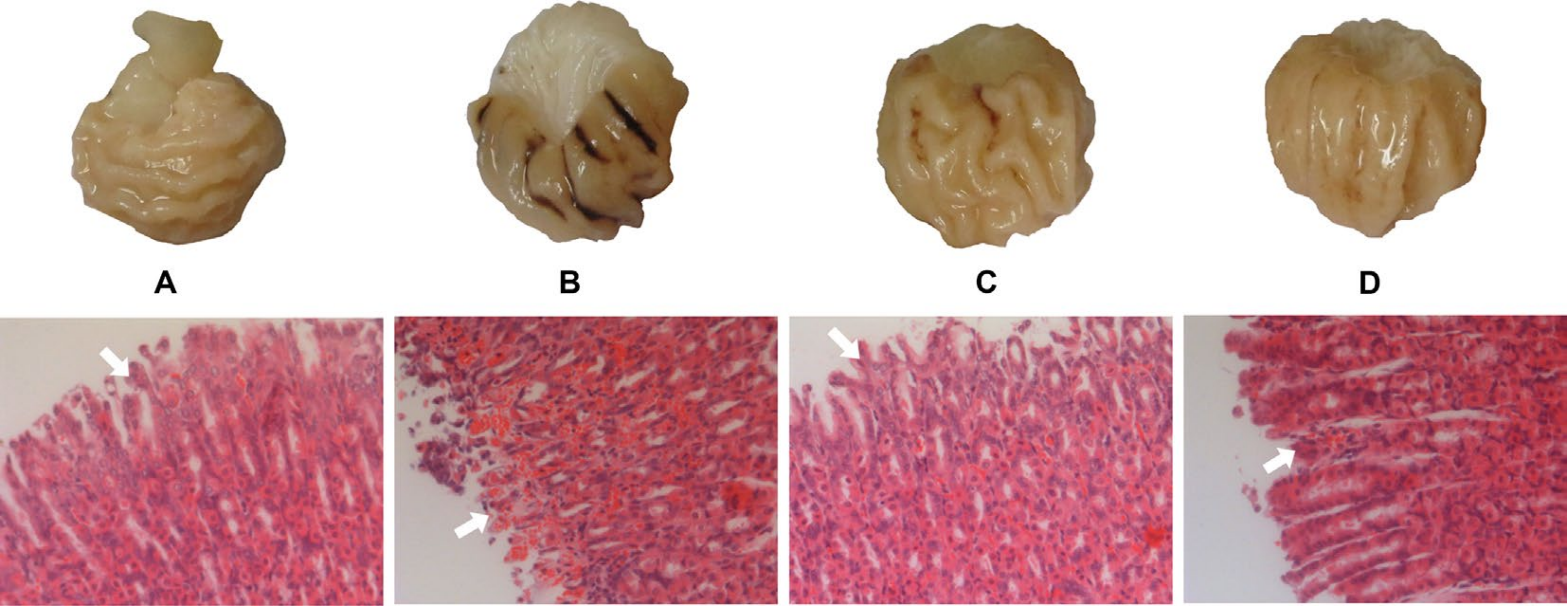
Macroscopic appearance and histological evaluation of gastric mucosa in rats with ethanol-induced gastric mucosal damage. **(A)** Normal control group; **(B)** model group treated with absolute ethanol; **(C)** *Gardenia* Fructus (GF) group at a dose of 4.5 g·kg^−1^; **(D)** carbonized GF (GFC) group at a dose of 4.5 g·kg^−1^. There were six rats in each group. Gross observation revealed that the area of hemorrhagic gastric mucosa was markedly decreased in the GF and GFC groups. Moreover, histological analysis showed that GF and GFC exerted a protective effect on mucosa (white arrow) of rats with ethanol-induced gastric mucosa damage (hematoxylin and eosin staining magnification 10 × 20).

The gastric mucosa of rats in the NC group was not hemorrhagic and the appearance was normal. However, there were differences between the M, GF, and GFC groups in the hemorrhagic lesions produced by administration of absolute ethanol. Gross observation revealed that the area of hemorrhagic gastric mucosa was decreased in the GF and GFC groups. In the NC group, there were no injuries observed. The administration of absolute ethanol resulted in a significant increase (*P* < 0.01) in hemorrhagic area (94.83 ± 20.27 mm^2^) compared with the NC group. This indicated that the model was successfully established. Moreover, the ulcer area in the GF (51.63 ± 26.72 mm^2^) and GFC (44.08 ± 32.34 mm^2^) groups was significantly reduced in comparison with the M group (**Figure 1**). Microscopic observation of H&E-stained sections showed that administration of absolute ethanol induced gastric mucosal segmental hemorrhage, edema, and lymphocytic hyperplasia in rats of the M group. Gastric mucosa with loss of superficial gastric mucosal glands cell and few hemorrhagic spots were observed after treatment with GF (**Figure 1**). In the GFC group, apparently normal mucosa with normal gastric mucosa glands and few hemorrhagic spots were observed (**Figure 1**). The rank-sum test results of the histological changes showed that GF reduced damage to the gastric mucosal after administration of absolute ethanol to rats (*P* = 0.241). In addition, the GFC exerted a protective effect on the stomach mucosal in rats of the GFC group versus the M group (*P* = 0.005) (**Table S2 of Supplement**).

#### Antioxidant Activity of IL-6 and NO

The results of the NO and IL-6 analysis are shown in **Table 1**. Treatment of rats with absolute ethanol caused a significance increase in the serum level of NO (89%, receptively (*P* = 0.000) compared with that observed in the NC group. Treatment of these rats with GF and GFC resulted in a decrease in the serum level of NO (22.6% and 6.8%, receptively) (*P* = 0.066 and *P* = 0.430, compared) compared with that reported in the model rats. The level of IL-6 in the serum of animals in the M group was significantly increased (51.9%, receptively) (*P* = 0.001) compare with that reported in the NC group. Notably, rats with ethanol-induced gastric ulcers treated using GF and GFC exhibited a non-significant decrease in the levels of IL-6 (10.7% and 11.4%, receptively).

**Table 1:** Effect of *Gardenia* Fructus (GF) and carbonized GF (GFC) on the level of nitric oxide (NO) and interleukin 6 (IL-6) in the serum of rats in the ethanol-induced acute gastric ulceration model.

	NC	M	GF	GFC
NO (야μmol·L^−1^)	16.23 ± 5.11	30.59 ± 5.79**	23.68 ± 6.17	28.51 ± 2.59
IL-6 (*ng*·L^−1^)	95.45 ± 13.12	144.95 ± 18.92**	129.45 ± 31.95	128.47 ± 45.1

Data are mean ± SD (n = 6), *P < 0.05, **P < 0.01 versus NC; ^#^P < 0.05, ^##^P < 0.01 versus M.

### Metabonomic Results

#### PCA

**Figure 2:**
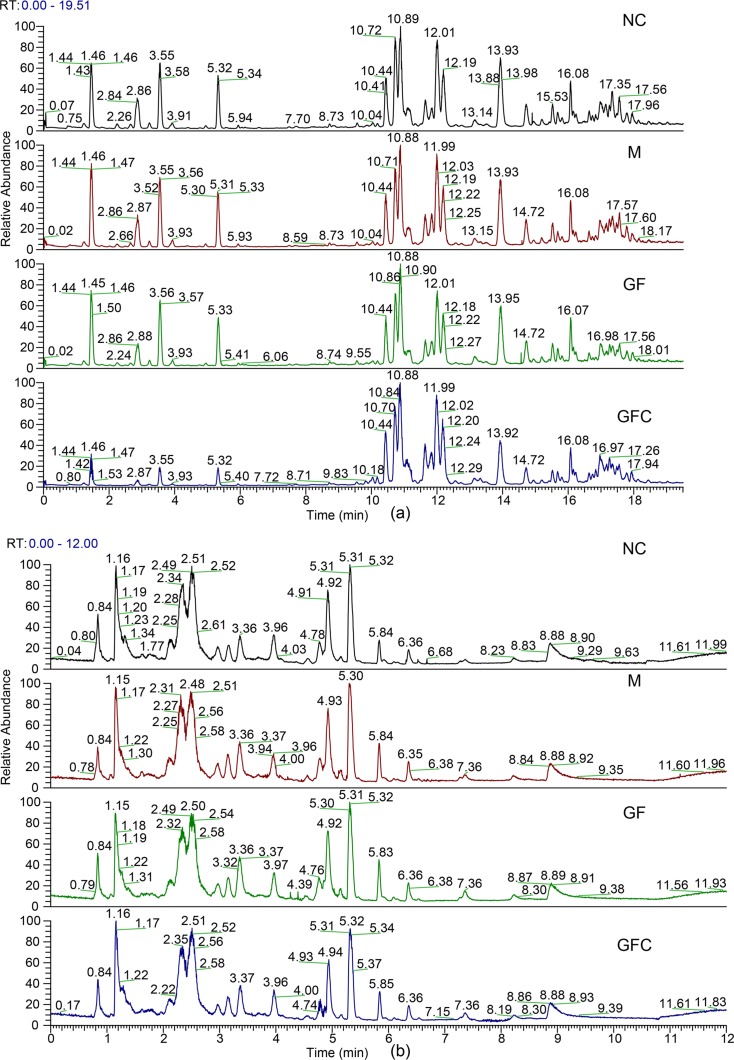
Total ion chromatograms of the normal control (NC), model (M), GF, and GFC groups in the C18/UHPLC/MS **(A)** and HILIC/UHPLC/MS **(B)** system with positive mode.

**Figure 3:**
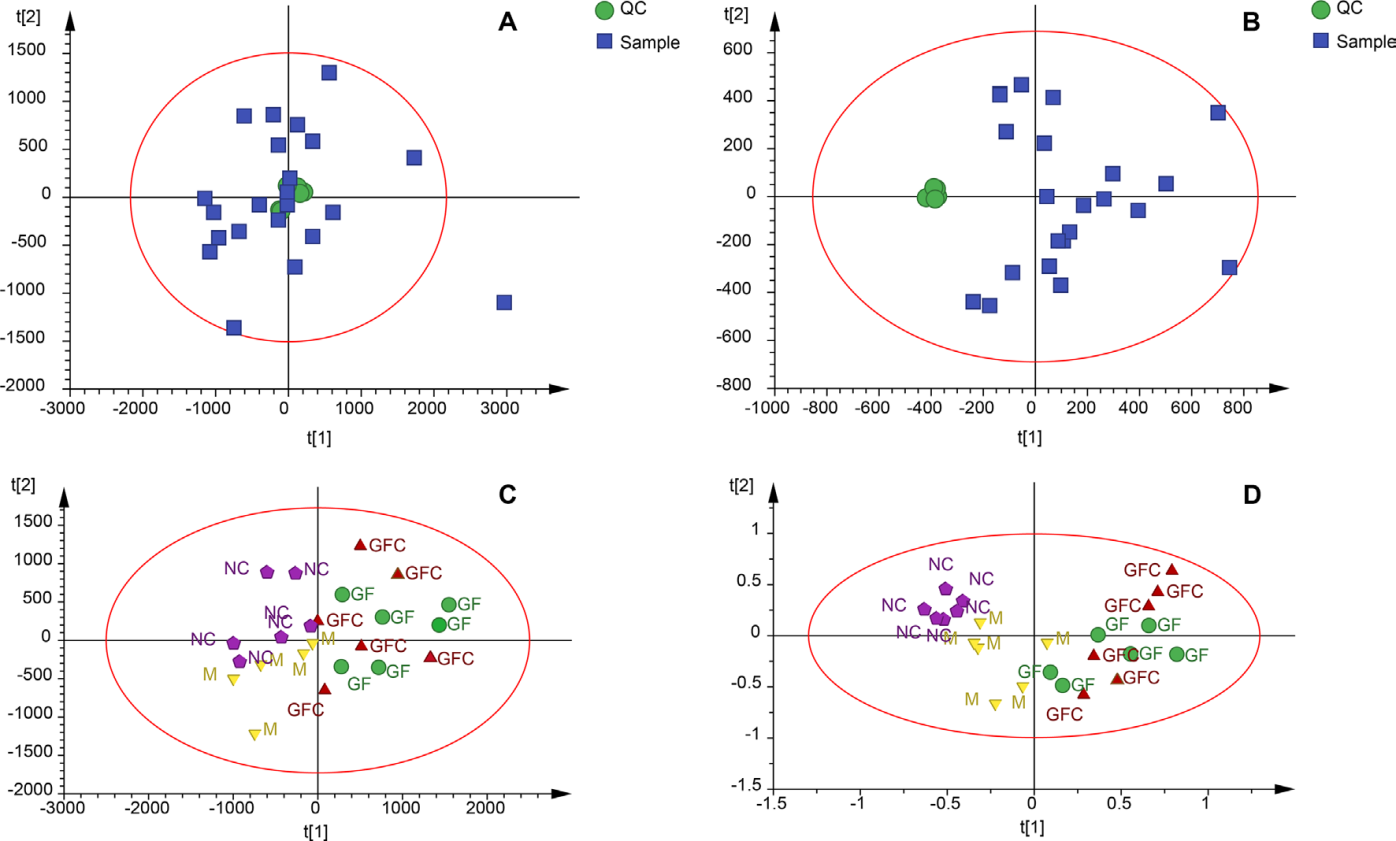
Quality control samples (QCs) evaluation and principal components analysis (PCA) plots in the HILIC and C18 separation modes. **(A)** QCs in the C18/UHPLC/MS system; **(B)** QCs in the HILIC separation mode; **(C)** PCA plot of all samples in the C18/UHPLC/MS system; **(D)** PCA plot of all samples in the HILIC separation. QCs clustered together in both the C18 and HILIC systems. PCA plots showed that the GF and GFC groups were separated from the M group.

Metabolomics data were obtained from the total ion current in HILIC and C18 separation. (**Figure 2**) The QCs clustered together and there was no variable with a CV beyond 15% in both the HILIC and C18 separation modes. Hotelling’s T2 observation using the SIMCA-p software determined that there was no outlier in both the HILIC and C18 separation modes. In the HILIC separation, the model was autofitted in four components with 52.3% of the variables explained in all groups. The score plot demonstrates that there was no outlier and the rats treated with absolute ethanol tended to move to the right in the HILIC separation. Meanwhile, the ethanol-induced gastric ulcer rats treated with GF and GFC clustered in same trends and separated with M group (**Figure 3**). This result indicated that the OPLS-DA model can be used to determine the feature metabolites of GF or GFC. In the C18 separation mode, all groups were autofitted in three components with 65.3% of the variables explained. The score plot of C18 showed that the M group was separated from the NC group, and rats tended to move to the right after treatment with GF or GFC.

#### OPLS-DA

**Figure 4:**
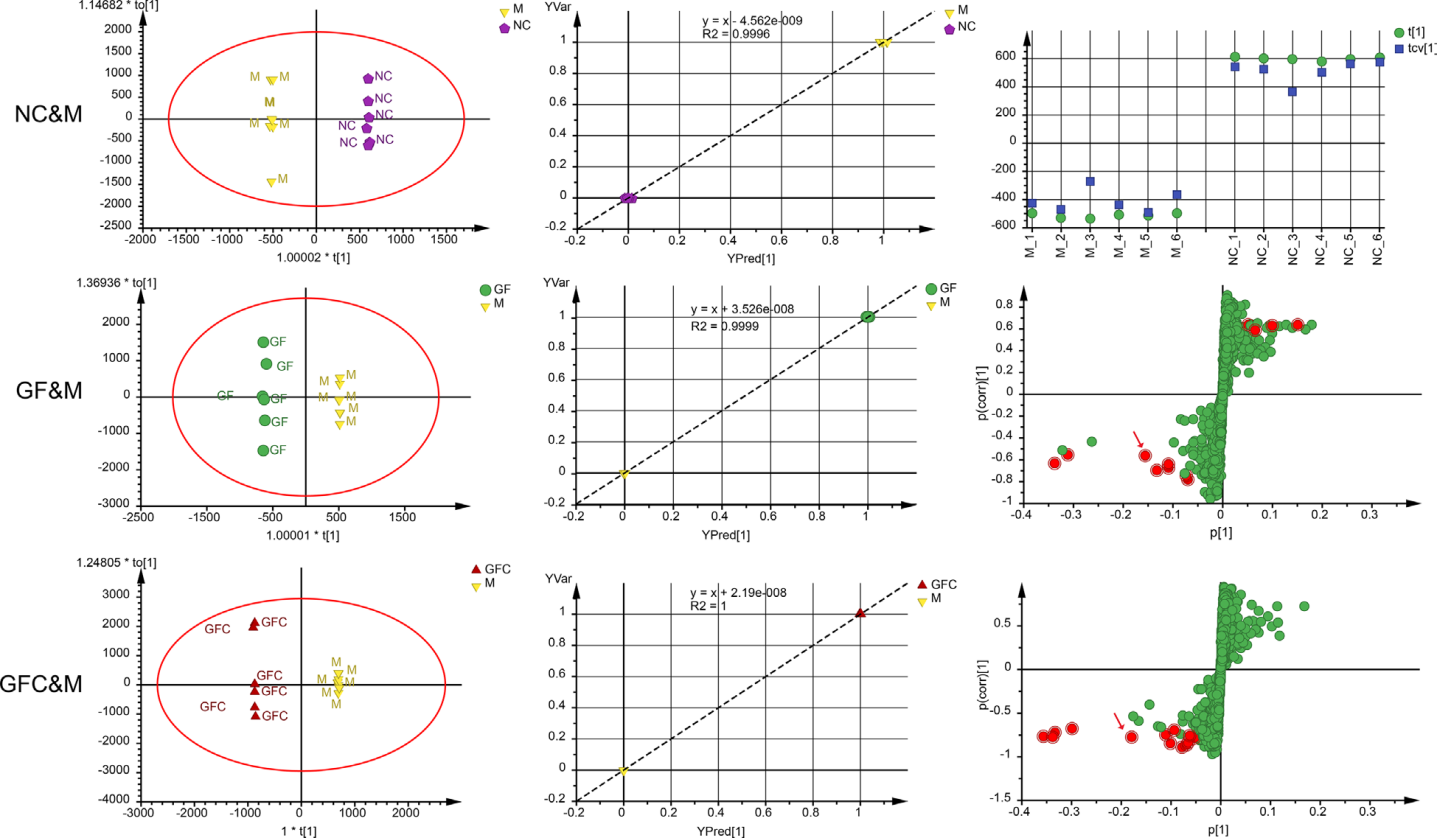
Score plots, observation versus predicted plots, coefficient of variation (CV)-plot (NC&M), and s-plots using orthogonal partial least squares discriminant analysis (OPLS-DA) in the C18/UHPLC/MS system. Red points (red arrows) in s-plots show the putative ion features with 95% confidence.

In the C18 separation mode, a 1 + 4-component OPLS model between the NC and M groups was obtained. Of note, 22.2% of the variations in the data were due to the ethanol-induced treatment. The uncorrelated information accounted for 59.3% of the variation in the data. The plot of observation versus predicted showed a complete separation of the M and NC groups. Each cluster of the NC and M groups overlapped within a 0.5 mark on the *X* axis. The CV-score plot of the M and NC groups showed that all samples were predicted to their own class during cross validation. The above results indicated that the OPLS-DA model of the M and NC groups was not over fitted and spurious. In the C18 separation mode, the OPLS-DA yielded six (1 predictive + 5 orthogonal) components with R2X = 0.89 and Q2 = 0.82 between the GF and M groups. In addition, 21.3% of the variations in the data was related to the treatment with GF. The plot of observed versus predicted based on the six components showed two clusters of GF and M focus on their own class which indicated that this was a stable model. A 1 + 6-component OPLS model between the GFC and M groups in the C18 separation mode was also obtained. In total, 98.1% of variations were interpreted of the total variation in GFC and M, and 31.1% of variations correlated with GFC treatment. The S-plot was evaluated together with the 95% confidence intervals to identify potential biomarkers after treatment (**Figure 4**). There were 12 and 13 feature ions of the GF and GFC groups had re-call regulation effect by comparing with rats treated with absolute ethanol (*P* < 0.05).

**Figure 5:**
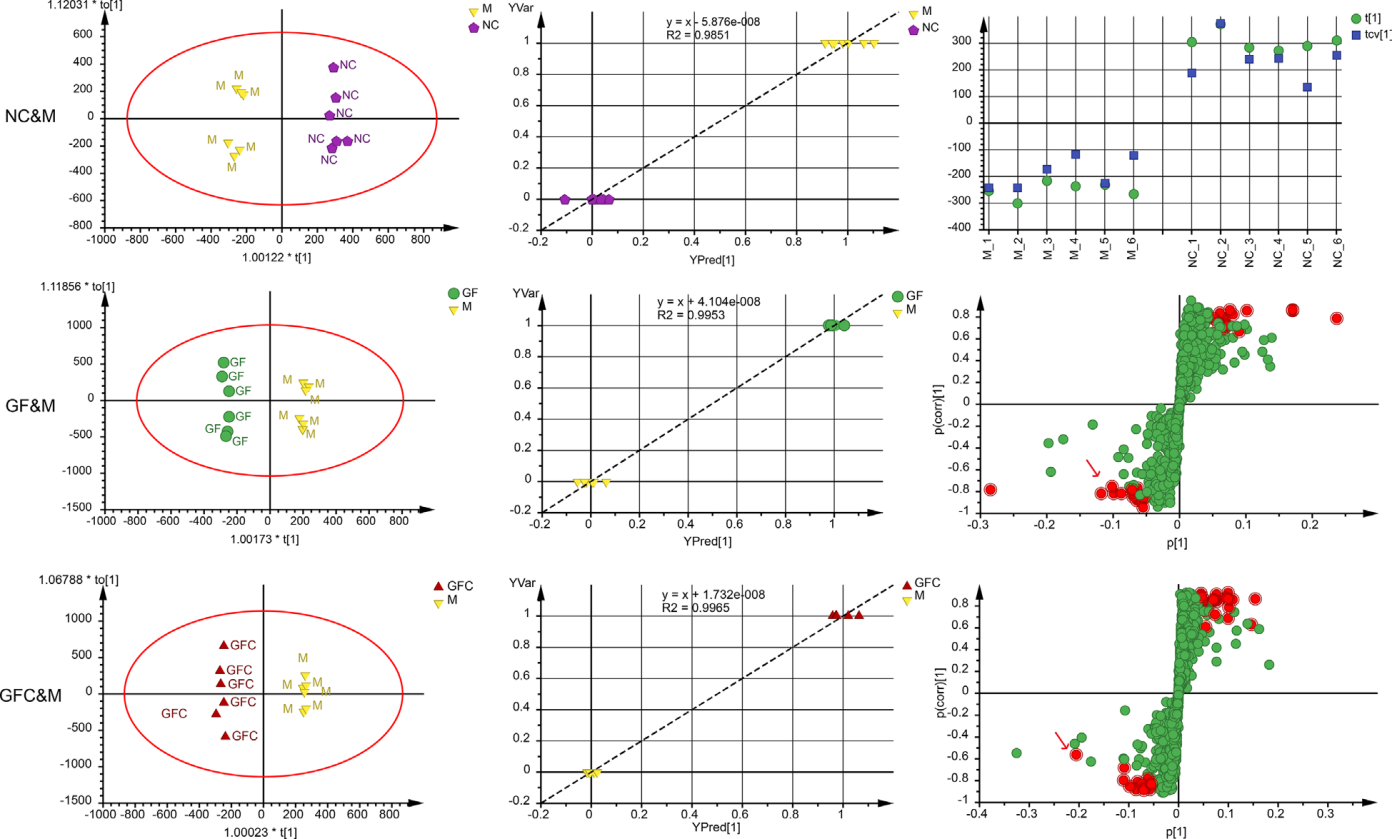
Score plots, observation versus predicted plots, CV-plot (NC&M), and s-plots using OPLS-DA in the HILIC/UHPLC/MS system. Red points (red arrows) in s-plots show the putative ion features with 95% confidence.

Of note, 72.3% of the variation in the data was explained using a 1 + 3-component OPLS model in the HILIC separation mode between the M and NC groups with Q2 = 0.844; 30.4% of the variation was attributed to the treatment with absolute ethanol. The cross-validated score plot showed that all samples were distributed at their own class. The plot of observed versus predicted showed that the M and NC groups clustered at their own class and besides the regression line. These results showed that the fitted OPLS model between the NC and M groups was stable. A 1 + 2-component OPLS model of M and GF was fitted with R2X = 0.571, Q2 = 0.771, and 17.0% of the variation in the data was attributed to treatment with GF. The plot of observation versus predicted showed that the OPLS model fitted of M and GF was stable. Of note, 20.1% of variation in the data between the M and GFC groups was correlated to treatment with GFC based on a 1 + 3-component OPLS model. The plot of observation versus predicted showed a complete separation of the M and GFC groups with all samples overlapping within a 0.5 mark on the *X* axis. Finally, there were 21 and 19 feature ions of the GF and GFC groups captured from the s-plot (**Figure 5**).

### Identification of Feature Metabolites

**Figure 6:**
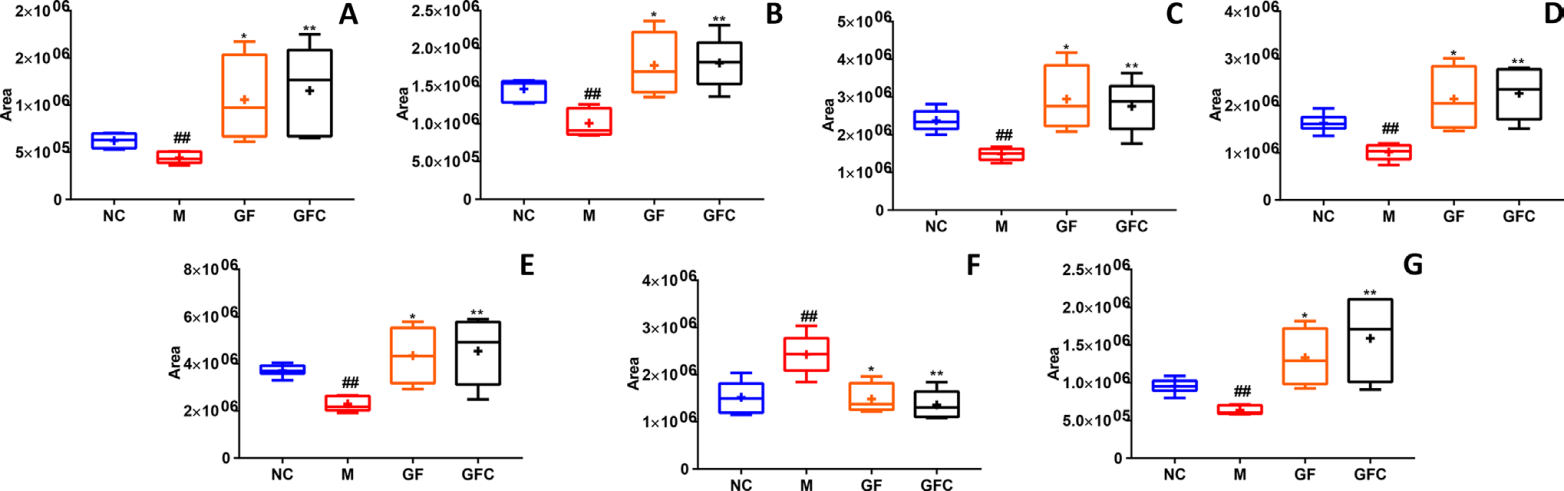
The relative content of common metabolites in the GF, GFC, and NC groups versus the M group. ^##^ *P* < 0.01, versus the NC group; *P < 0.05, versus the M group; **p < 0.01, versus the M group. **(A)** Trans-hexadec-2-enoyl carnitine; **(B)** 2-hydrohexadecanoylcarnitine; **(C)** 3-hexenoic acid; **(D)** lioelaidyl carnitine; **(E)** L-palmitoylcarnitine; **(F)** methyl nicotinate; **(G)** tetradecanoylcarnitine. Plots showed that seven common feature metabolites of the GF and GFC groups were significantly recalled using a multiple *t*-test. Besides, these seven common metabolites were significantly recalled in the GFC group with a false discovery rate (FDR) below 1%, receptively.

A total of 19 putative biomarkers were identified based on S-plot results according accurate *m/z* by matching to the HMDB database in the HILIC and C18 separation modes of GF and GFC. Both the GF and GFC groups showed 10 feature metabolites correlated with ethanol-induced gastric ulcer in rats after treatment with drug (**Table 2**). A multiple *t*-test was performed for the common metabolites; seven of those were significantly recalled in the GFC group with a false discovery rate (FDR) below 1% (**Figure 6**). Besides the common feature metabolites, 3-hydroxy-9-hexadecenoylcarnitine, L-glutamine, argininic acid, sphingomyelin (d18:0/16:1(9Z)), and cholesterol ester (20:4(8Z,11Z,14Z,17Z)) were putative metabolites of the GF group that exhibited a significantly call-backed effect. Notably, PC(18:1(9Z)/18:1(9Z)), L-histidine, and PC(22:4(7Z,10Z,13Z,16Z)/14:0) were unique metabolites of GFC that were significantly up-regulated after treatment with GFC in rats with ethanol-induced gastric ulcer (**Table 2**).

**Table 2:** Putative biomarkers in rats treat with GF and GFC with HILIC/C18-UHPLC/MS.

No.	Name	t_R_/min	*m/z*	Adduct	Formula	Separation	HMDB	Recall effect	Group	Della (ppm)
1	2-Hydroxyhexadecanoylcarnitine	1.28	425.3502	M	C_25_H_47_NO_4_	HILIC	HMDB13337	↑	GF/GFC	1
2	L-Palmitoylcarnitine	1.41	399.3345	M	C_23_H_45_NO_4_	HILIC	HMDB00222	↑	GF/GFC	0
3	Linoelaidyl carnitine	1.41	424.3418	M+H^+^	C_25_H_45_NO	HILIC	HMDB06461	↑	GF/GFC	1
4	trans-Hexadec-2-enoyl carnitine	1.42	398.3262	M+H^+^	C_23_H_43_NO_4_	HILIC	HMDB06317	↑	GF/GFC	1
5	Tetradecanoylcarnitine	1.45	371.3033	M	C_21_H_41_NO_4_	HILIC	HMDB05066	↑	GF/GFC	1
6	3-Hexenoic acid	4.92	132.1019	M+NH_4_^+^	C_6_H_10_O_2_	HILIC	HMDB31501	↑	GF/GFC	0
7	L-Valine	4.93	118.0864	M+H^+^	C_5_H_11_NO_2_	HILIC	HMDB00883	↓	GF/GFC	1
8	Methyl nicotinate	5.15	138.0550	M+H^+^	C_7_H_7_NO_2_	HILIC	HMDB29806	↓	GF/GFC	0
9	SM(d18:0/18:1(11Z))	11.70	730.5982	M	C_41_H_83_N_2_O_6_P	C18	HMDB12088	↑	GF/GFC	1
10	PC(20:0/16:1(9Z))	11.99	810.5992	M+Na^+^	C_44_H_86_NO_8_P	C18	HMDB08266	↑	GF/GFC	1
11	3-Hydroxy-9-hexadecenoylcarnitine	1.67	414.3212	M+H^+^	C_23_H_43_NO_5_	HILIC	HMDB13333	↓	GF	0
12	LysoPC(24:1(15Z))	2.00	605.4418	M	C_32_H_64_NO_7_P	HILIC	HMDB10406	↓	GF	0
13	L-Glutamine	6.35	147.0763	M+H^+^	C_5_H_10_N_2_O_3_	HILIC	HMDB00641	↓	GF	1
14	Argininic acid	6.54	175.0957	M	C_6_H_13_N_3_O_3_	HILIC	HMDB03148	↓	GF	0
15	SM(d18:0/16:1(9Z))	10.41	702.5664	M	C_39_H_79_N_2_O_6_P	C18	HMDB13464	↑	GF	2
16	CE(20:4(8Z,11Z,14Z,17Z))	17.95	672.5841	M	C_47_H_76_O_2_	C18	HMDB10371	↑	GF	2
17	PC(18:1(9Z)/18:1(9Z))	1.15	786.5995	M+H^+^	C_44_H_84_NO_8_P	HILIC	HMDB00593	↑	GFC	3
18	L-Histidine	7.34	155.0695	M	C_6_H_9_N_3_O_2_	HILIC	HMDB00177	↑	GFC	0
19	PC(22:4(7Z,10Z,13Z,16Z)/14:0)	10.72	781.5601	M	C_44_H_80_NO_8_P	C18	HMDB08623	↑	GFC	2

‘↑’ stands for up-regulation; ‘↓’ stands for down-regulation.

### Pathway Analysis

**Figure 7:**
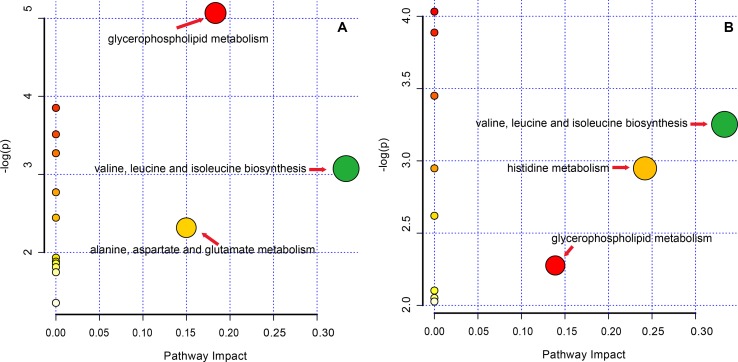
Pathway analysis in rats with ethanol-induced gastric lesions treated with GF and GFC. **(A)** (GF group); **(B)** (GFC group).

The altered pathway of the GF and GFC groups was enrichment by KEGG code with MetaPA, 16 and 12 pathways of the GF and GFC groups assigned by full featured metabolites in MetaPA (**Figure 7**). The significantly changed metabolites mainly hits C00157, C00183, C00135, and C00064 in glycerophospholipid metabolism, valine, leucine and isoleucine biosynthesis, alanine, aspartate and glutamate metabolism, and histidine metabolism (**Table S3** and **S4 of Supplement**). These metabolic pathways were found to disrupt in rats with ethanol-induced gastric lesion. Treatment with GF restored the pathway of valine, leucine and isoleucine biosynthesis (impact = 0.333), glycerophospholipid metabolism (impact = 0.183), and alanine, aspartate, and glutamate metabolism (impact = 0.150). Treatment with GFC restored valine, leucine and isoleucine biosynthesis (impact = 0.333), histidine metabolism (impact = 0.242), and glycerophospholipid metabolism (impact = 0.139).

## Discussion

Previous studies showed that the occurrence of gastrointestinal hemorrhage lesions increased 1 h after administration of ethanol (Szabo et al., 1985; Murakami et al., 1988). In this study, both macroscopic and histological evaluations of gastric lesions showed that ethanol-induced gastric hemorrhage lesions and damage to gastric mucosa in rats were reduced after treatment with GF and GFC versus the M group. In addition, treatment with GFC prevented gastrointestinal damage in rats with ethanol-induced gastric ulcers in both macro and micro.

Our study also found that the level of NO was increased in rats with ethanol-induced ulcers. However, this trend was reversed after the administration of GF and GFC. Accumulated evidence has shown that NO plays a double-edged role in mucosal integrity *via* numerous functions (Schmidt and Walter, 1994; Calatayud et al., 2001). It is established that NO plays a protective role in physiological conditions. However, following damage of the mucosa, the functions of NO become complex. Studies have shown that unregulated release of high levels of NO may induce mucosal injury and susceptibility of gastric mucosa under portal-hypertensive conditions (Lopez-Belmonte et al., 1993; Ohta et al., 1997). Meanwhile, using metabolomics we found that the expression of argininic acid, which synthesized NO, was down-regulated in the GF group. This finding indicated that the protective effect exerted by GF and GFC in these rats may occur through the reduction of argininic acid expression to protect gastric mucosal integrity.

**Figure 8:**
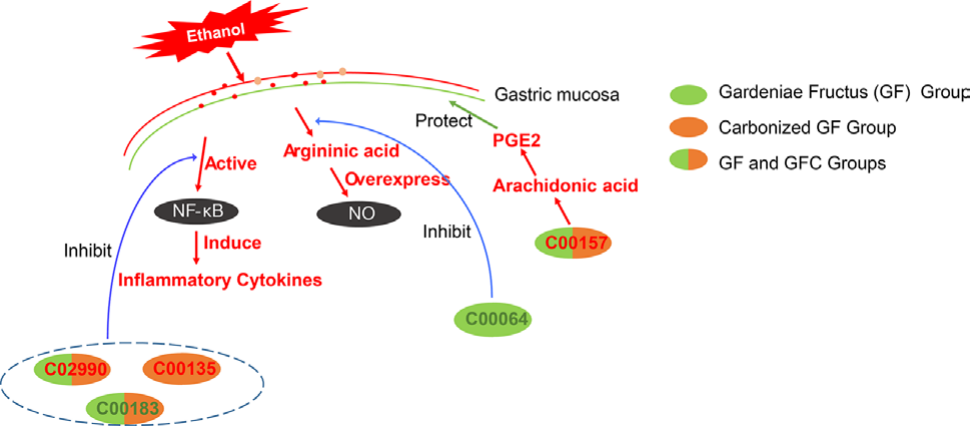
The putative gastroprotective mechanism of GF and GFC in rats with ethanol-induced gastric lesions. GF and GFC reduce the expression of NO and release of inflammatory cytokines, and induce prostaglandin E2 (PGE2) to ameliorate gastric mucosa lesions in rats. C02990 and C00157 (red text) refer to L-palmitoylcarnitine and phosphatidylcholines which were significantly upregulated in the GF and GFC groups. C00183 (green text) refers to L-valine which was significantly downregulated in the GF and GFC groups. C00064 (green text) refers to L-glutamine which was down-regulated in the GF group. C00135 (red text) refers to L-histidine which was markedly upregulated in the GFC group.

The identified common feature metabolites between GF and GFC were C00157, C02990, C00550, and C00183. GF and GFC present on 11 same pathways for the prevention of gastric mucosal damage. The results of the pathway analysis showed that both GF and GFC restore the biosynthetic pathway of valine, leucine, and isoleucine. The feature metabolite, L-valine (C00183) with downstream effect after treatment with GF and GFC in rats with ethanol-induced gastric hemorrhage. L-valine belongs to branched-chain amino acids which play an important role in inflammation (De Simone et al., 2013). Studies showed that accumulated branched-chain amino acids actives the NF-κB pathway and induces the release of inflammatory cytokines (Zhenyukh et al., 2017). In this study, a cytokine assay showed that the expression of IL-6 was deregulated by GF and GFC. The results suggested that GF and GFC may regulate the expression of L-valine to suppress inflammatory cytokines such as IL-6, resulting in an anti-inflammatory effect (**Figure 8**).

Furthermore, both GF and GFC up-regulated the expression of phosphatidylcholine (C00157) which synthesizes linoleic acid and arachidonic acid with secretory phospholipase A2 (PLA2G). In addition, the high levels of linoleic acid and arachidonic acid may suppress the gastric mucosa against injury related to produce the prostaglandin E2 (PGE2) (Tarnawski et al., 1987; Schepp et al., 1988). Evidence suggested that endogenous PGE2 plays an important role in gastroprotection and protects the gastric tract mucosa from damage (Wilson, 1991; Synnerstad and Holm, 1998).

Previous studies showed that palmitoylcarnitine inhibits the activation of protein kinase C (PKC) (Nakadate and Blumberg, 1987), and certain PKC inhibitors exert anti-inflammatory and analgesic effects (Selvatici et al., 2010). In our study, the expression of palmitoylcarnitine (C02990) was up-regulated after treatment with GF and GFC. This result indicated that GF and GFC may enhance the expression of palmitoylcarnitine to inhibit PKC activation, leading to an anti-inflammatory effect.

The results of the pathway analysis showed that the biosynthesis of valine, leucine, and isoleucine, and the metabolism of glycerophospholipids were the two main pathways regulated by the administration of GF and GFC. Both GF and GFC exert the same regulatory effect on the biosynthesis of valine, leucine, and isoleucine. In contrast, the impact on the metabolism of glycerophospholipids differed between GF and GFC, as shown by the numerous feature metabolites. Besides, the main pathway was converted from alanine, aspartate, and glutamate metabolism in the GF group to histidine metabolism in the GFC group. The results showed that the levels of both L-glutamine (C00064) and argininic acid—involved in alanine, aspartate, and glutamate metabolism—decreased significantly in the GF group. This finding indicated that GF may inhibit the expression of L-glutamine to suppress argininic acid, preventing damage to the gastric mucosa from overexpressed NO (**Figure 8**).

In contrast, GFC changed the main pathway from aspartate and glutamate metabolism in the GF group to histidine metabolism. In this study, a decreasing level of histidine (C00135) was observed in rats with ethanol-induced gastric lesions. This trend was significantly reversed in the GFC group. Strong evidence showed that low levels of histidine were associated with inflammation, insulin resistance, and chronic gastrointestinal disease (Watanabe et al., 2008; Sakai et al., 2018). Clinical studies showed that histidine exhibits anti-inflammatory, antioxidant, and radical-scavenging activity, and may inhibit IL-8 release and NF-κB activation (Decker et al., 2001; Lee et al., 2005; Hasegawa et al., 2012). These findings indicated that GFC may induce the synthesis of histidine to prevent the secretion of inflammatory cytokines and oxidative damage to gastric mucosal gland cells by ethanol (**Figure 8**).

## Conclusions

Treatment with GF and GFC restored the biosynthesis of valine, leucine, and isoleucine, metabolism of glycerophospholipids. Moreover, histological evaluation revealed that heat processing of GF to create GFC enhanced the gastric mucosa protective effect. Furthermore, heat processing converted the main pathway from alanine, aspartate, and glutamate metabolism, associated with GF, to histidine metabolism, associated with GFC. GF and GFC ameliorated gastric mucosa lesions in rats *via* a reduction in the expression of NO and secretion of inflammatory cytokines, and induction of PGE2.

## Ethics Statement

This investigation was approved by the Ethics Committee on the Welfare of Laboratory Animals of Institute of Chinese Materia Medica of China Academy of Chinese Medical Sciences (no. 20172006). All applicable international, national, and/or institutional guidelines for the care and use of animals were followed.

## Author Contributions

CZ, XZ, and YW designed the research; YD, XL, QW, GW, and DL performed the experiments. XG, DY and YM performed the histological experiments. XZ analyzed the data and drafted the manuscript; CZ and YW critically revised the manuscript.

## Funding

Financial support was provided by the National Natural Science Foundation of China (nos. 81873010, 81473356, 81703708, and 81173553), National Project for Standardization of Traditional Chinese Medicine (no. ZYBZH-Y-SH-38 and no. ZYBZH-Y-JIN-34), National Science and Technology Special Project for New Drugs Innovation (no. 2014ZX09304307001), and the Basic Research Program of the China Academy of Chinese Medical Sciences (no. ZZ2014053).

## Conflict of Interest Statement

The authors declare that the research was conducted in the absence of any commercial or financial relationships that could be construed as a potential conflict of interest.
