# Supplementary material for: Treatment Mechanism of Gardeniae Fructus and Its Carbonized Product Against Ethanol-Induced Gastric Lesions in Rats
Source: Front Pharmacol. 2019 Jul 3;10:750. doi: 10.3389/fphar.2019.00750 (PMC6616308; doi:10.3389/fphar.2019.00750)
Supplement: Supplementary file 1 [file DataSheet_1.doc]

Treatment Mechanism of Gardeniae Fructus and Its Carbonized Product Against Ethanol-induced Gastric Lesions in Rats

Xue ZHANG1,[[1]](#footnote-2)#, Yun WANG^1,#^, Xiao-Qing LI^1,2^, Ye-jia DAI^1,3^, Qing-hao WANG^1,2^, Guo-you WANG^1,2^, De-peng LIU^1,3^, Xue-zhu GU^1^, Ding-rong YU^1^, Yin-lian MA^1^, Cun ZHANG1,2,3[[2]](#footnote-3)*

*^1^* *Institute of Chinese Materia Medica, China Academy of Chinese Medical Sciences, Beijing 100700, China; ^2^ College of Pharmacy, Henan University of Chinese Medicine, Zhengzhou 450046, China ^3^College of Pharmacy, Anhui University of Chinese Medicine, Hefei 230038, China*

**Determination of iridoids and crocins of GF and GFC freeze-dried powder**

To analyse the major active constituents and assess the quality of Gardeniae Frutus (GF) and carbonized GF (GFC), they were analysed by HPLC. The typical chromatograms of the GF and GFC were presented in Figure 1. Six peaks of GF were identified by chemical standards which were gardoside; genipin 1- gentiobioside (G1), geniposide (G2), p-coumaroylgenipin gentiobioside (G3), crocin I and crocin II (structure shown in Figure 2). The results showed the contents of six constituents of GF and GFC were decreased in the heat process. Crocins were decreased sharply by the heat process and crocin II were failed to determination in GFC (Table 1). However, the content of geniposide in GF and GFC freeze-dried fulfilled the demand of *Chinese Pharmacopeia.*

Table 1 The content of six constituents among GF and GFC freeze-dried powder with HPLC (mg∙g^-1^).

|  | Gardoside | G1 | G2 | G3 | Crocin I | Crocin II |
| --- | --- | --- | --- | --- | --- | --- |
| GF | 7.56 | 23.51 | 46.61 | 12.53 | 8.05 | 0.34 |
| GFC | 1.67 | 16.71 | 29.75 | 5.38 | 0.05 | - |

‘-’ stands for constituent failed to quantity.


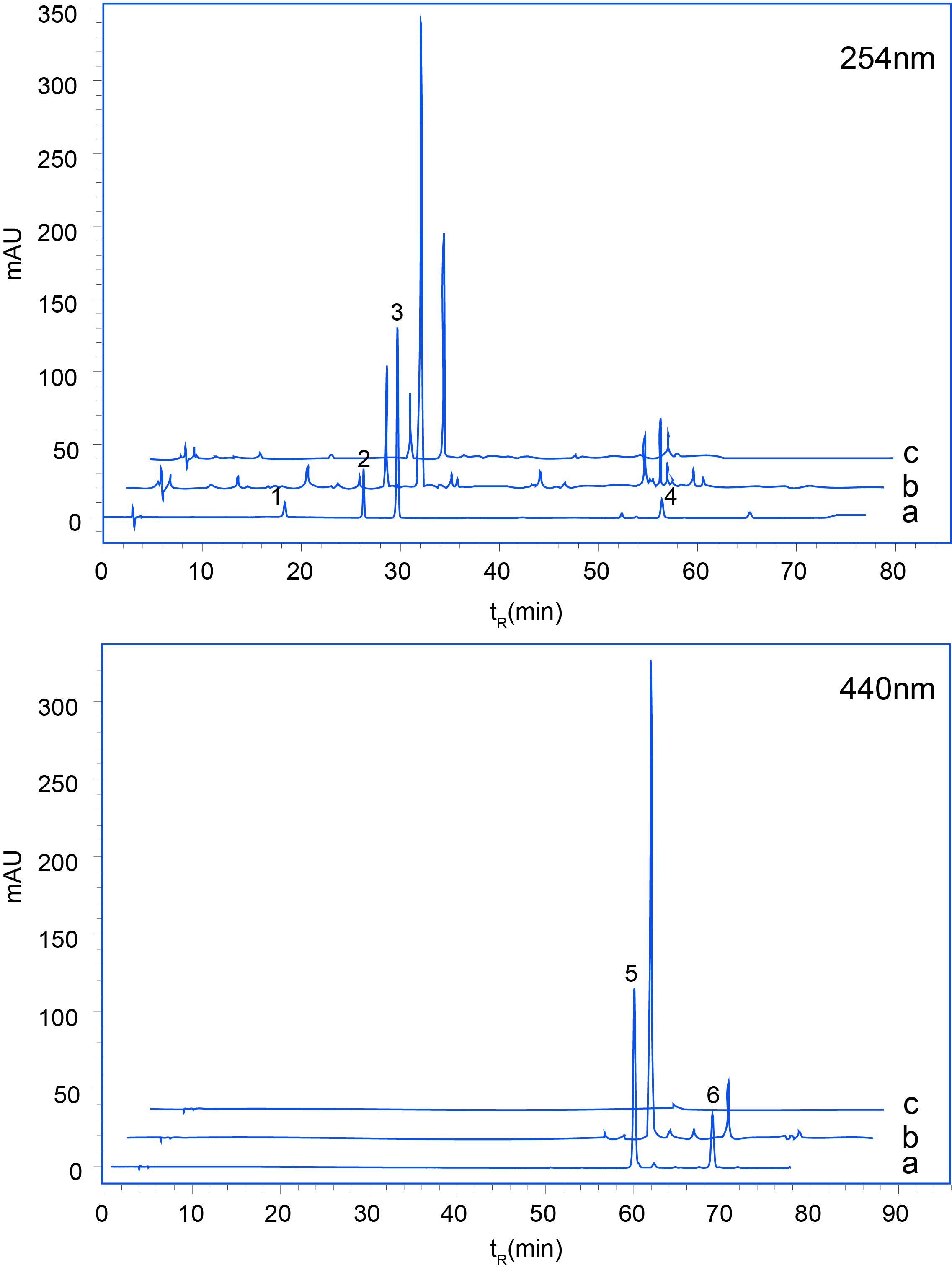


Figure 1 Typical chromatographs of GF and GFC recorded at 254nm and 440nm.a stands for reference standards, b stands for GF freeze-dried powder, c stands for GFC freeze-dried powder. In chromatographs, 1 was gardoside, 2 was G1, 3 was G2, 4 was G3, 5 was crocin I and 6 was crocin II.

**gardoside**: (1S,4aS,6S,7aS)-6-hydroxy-7-methylidene-1-[(2S,3R,4S,5S,6R)-3,4,5-trihydroxy-6-(hydroxymethyl)oxan-2-yl]oxy-4a,5,6,7a-tetrahydro-1H-cyclopenta[c]pyran-4-carboxylic acid

**genipin 1- gentiobioside**: methyl (1S,4aS,7aS)-7-(hydroxymethyl)-1-[(2S,3R,4S,5S,6R)-3,4,5-trihydroxy-6-[[(2R,3R,4S,5S,6R)-3,4,5-trihydroxy-6-(hydroxymethyl)oxan-2-yl]oxymethyl]oxan-2-yl]oxy-1,4a,5,7a-tetrahydrocyclopenta[c]pyran-4-carboxylate

**geniposide**: methyl (1S,4aS,7aS)-7-(hydroxymethyl)-1-[(2S,3R,4S,5S,6R)-3,4,5-trihydroxy-6-(hydroxymethyl)oxan-2-yl]oxy-1,4a,5,7a-tetrahydrocyclopenta[c]pyran-4-carboxylate

**p-coumaroylgenipin gentiobioside**: (1S,4aS,7aS)-7-(hydroxymethyl)-1-[(2S,3R,4S,5S,6R)-3,4,5-trihydroxy-6-[[(2R,3R,4S,5S,6R)-3,4,5-trihydroxy-6-[[(E)-3-(4-hydroxyphenyl)prop-2-enoyl]oxymethyl]oxan-2-yl]oxymethyl]oxan-2-yl]oxy-1,4a,5,7a-tetrahydrocyclopenta[c]pyran-4-carboxylic acid

**crocin I**: bis[(2S,3R,4S,5S,6R)-3,4,5-trihydroxy-6-[[(2R,3R,4S,5S,6R)-3,4,5-trihydroxy-6-(hydroxymethyl)oxan-2-yl]oxymethyl]oxan-2-yl] (2E,4E,6E,8E,10E,12E,14E)-2,6,11,15-tetramethylhexadeca-2,4,6,8,10,12,14-heptaenedioate

**crocin II:** 1-O-[(2R,3R,4S,5S,6R)-3,4,5-trihydroxy-6-(hydroxymethyl)oxan-2-yl] 16-O-[(2S,3R,4S,5S,6R)-3,4,5-trihydroxy-6-[[(2R,3R,4S,5S,6R)-3,4,5-trihydroxy-6-(hydroxymethyl)oxan-2-yl]oxymethyl]oxan-2-yl] (2E,4E,6E,8E,10E,12E,14E)-2,6,11,15-tetramethylhexadeca-2,4,6,8,10,12,14-heptaenedioate


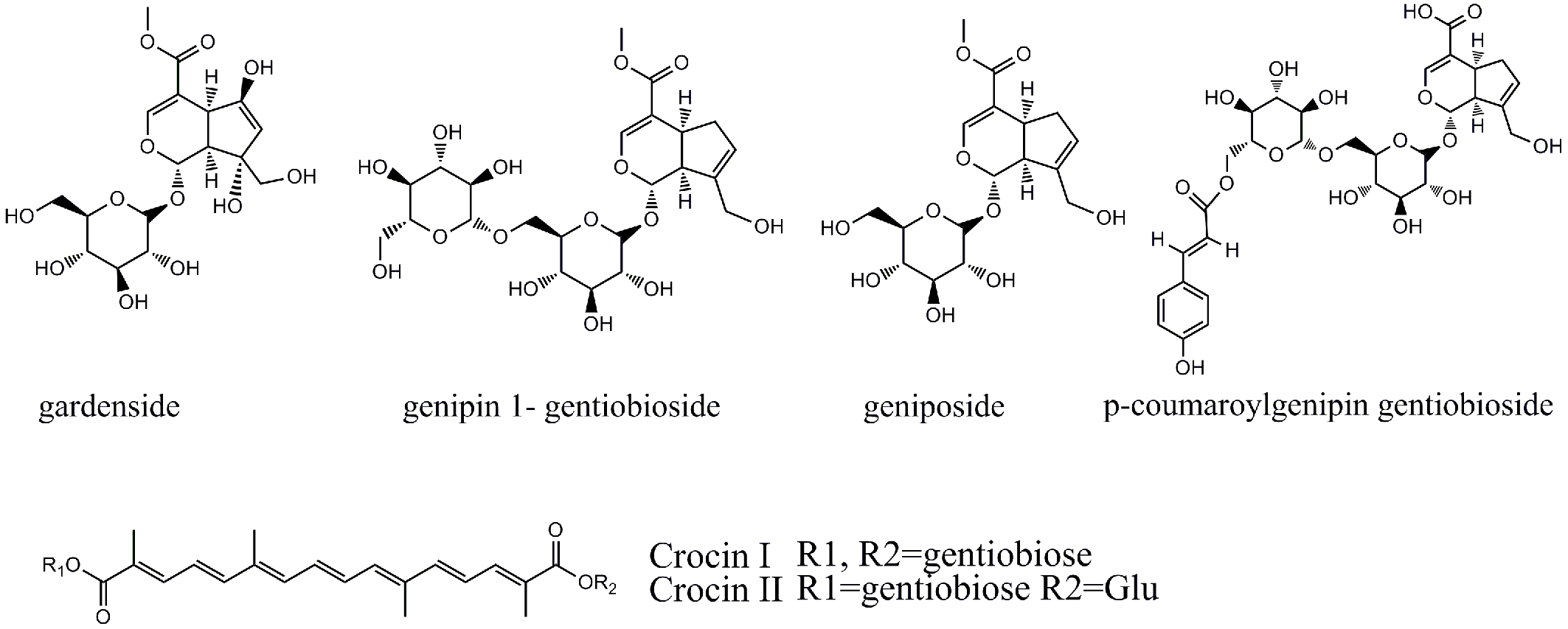


Figure 2 The structures of constituents were determined for this study.

**Histological evaluation of gastric lesions in microscopic**

Different damage degree of gastric mucosa in rats evaluated as follow, ‘+’-small mucosa segmental bleeding and normal musularis mucosa, ‘++’-mucosa segmental haemorrhage with mild lesion of musularis extrma, ‘+++’-gastric mucosa with segmental haemorrhage, musularis extrma edema with infiltration of inflammatory cells. Weighing frequency cased, and using rank sum test to compare the histopathology changes between M and groups treated with drug.

Table 2 Gastric mucosa damage evaluation in microscopic using rank sum test for GF and GFC versus M groups (n=6)

|  | + | ++ | +++ | P value |
| --- | --- | --- | --- | --- |
| M | 0 | 3 | 3 | - |
| GF | 0 | 5 | 1 | 0.241 |
| GFC | 5 | 1 | 0 | 0.005^**^ |

^**^P<0.01 versus M group

**Pathway analysis**

The altered pathway of GF and GFC group was enrichment by KEGG code with MetaPA, 16 and 12 pathways of GF and GFC group assigned by full featured metabolites in MetaPA. The feature metabolites hits on phosphatidylcholine (C00157), L-Glutamine (C00064), LysoPC(18:1(9Z)) (C04230), L-Valine (C00183), SM (C00550), L- Palmitoylcarnitine (C02990), L-Histidine (C00135) in KEGG according to their HMDB code. Results showed that GF focus on correcting the pathway of valine, leucine and isoleucine biosynthesis (impact=0.333), glycerophospholipid metabolism (impact=0.183), and alanine, aspartate and glutamate metabolism (impact=0.150). GFC centred on valine, leucine and isoleucine biosynthesis (impact=0.333), histidine metabolism (impact=0.242), and glycerophospholipid metabolism (impact=0.139).

Table 3 Summary of pathway analysis with MetaPA of GF group

| Pathway Name | -Log(p) | Impact | Hits |
| --- | --- | --- | --- |
| Glycerophospholipid metabolism | 5.0884 | 0.1833 | Phosphatidylcholine, LysoPC(18:1(9Z)) |
| D-Glutamine and D-glutamate metabolism | 3.8622 | 0.0000 | L-Glutamine |
| Linoleic acid metabolism | 3.6816 | 0.0000 | Phosphatidylcholine |
| Aminoacyl-tRNA biosynthesis | 3.4778 | 0.0000 | L-Glutamine,  L-Valine |
| alpha-Linolenic acid metabolism | 3.2814 | 0.0000 | Phosphatidylcholine |
| Nitrogen metabolism | 3.2814 | 0.0000 | L-Glutamine |
| Valine, leucine and isoleucine biosynthesis | 3.0843 | 0.3333 | L-Valine |
| Pantothenate and CoA biosynthesis | 2.7812 | 0.0000 | L-Valine |
| Sphingolipid metabolism | 2.4553 | 0.0000 | SM |
| Alanine, aspartate and glutamate metabolism | 2.3271 | 0.1498 | L-Glutamine |
| Arachidonic acid metabolism | 1.9427 | 0.0000 | Phosphatidylcholine |
| Valine, leucine and isoleucine degradation | 1.8922 | 0.0000 | L-Valine |
| Fatty acid metabolism | 1.868 | 0.0000 | L-Palmitoylcarnitine |
| Pyrimidine metabolism | 1.8215 | 0.0000 | L-Glutamine |
| Arginine and proline metabolism | 1.7561 | 0.0000 | L-Glutamine |
| Purine metabolism | 1.3629 | 0.0000 | L-Glutamine |

Table 4 Summary of pathway analysis with MetaPA of GFC group

| Pathway Name | -Log(p) | Impact | Hits |
| --- | --- | --- | --- |
| Linoleic acid metabolism | 3.8622 | 0.0000 | Phosphatidylcholine |
| Aminoacyl-tRNA biosynthesis | 3.8516 | 0.0000 | L-Histidine, L-Valine |
| alpha-Linolenic acid metabolism | 3.4609 | 0.0000 | Phosphatidylcholine |
| Nitrogen metabolism | 3.4609 | 0.0000 | L-Histidine |
| Valine, leucine and isoleucine biosynthesis | 3.2631 | 0.3333 | L-Valine |
| Pantothenate and CoA biosynthesis | 2.9586 | 0.0000 | L-Valine |
| Histidine metabolism | 2.9586 | 0.2419 | L-Histidine |
| Sphingolipid metabolism | 2.6306 | 0.0000 | SM |
| Glycerophospholipid metabolism | 2.2866 | 0.1389 | Phosphatidylcholine |
| Arachidonic acid metabolism | 2.1128 | 0.0000 | Phosphatidylcholine |
| Valine, leucine and isoleucine degradation | 2.0616 | 0.0000 | L-Valine |
| Fatty acid metabolism | 2.0370 | 0.0000 | L-Palmitoylcarnitine |

1. ^^ Xue ZHANG and Yun WANG contributed equally to this work. [↑](#footnote-ref-2)
2. ^^ To whom correspondence should be addressed.

   E-mail [zhc95@163.com](mailto:zhc95@163.com) (Cun ZHANG) [↑](#footnote-ref-3)
